# Dr. Gunning's Article

**Published:** 1868-04

**Authors:** 


*Dr. Gunning's Article.*—We wish to direct the especial attention of our readers to Dr. Thomas B. Gunning's Article "On the Physiological Action of the Muscles which control and influence the Lower Jaw," the first part of which appears in the present number of the Journal. The views advanced by Dr. G. are not only novel but are altogether original, and founded upon true physiological reasonings. They will no doubt, completely revolutionize the opinions hitherto obtained from our standard works upon this subject.

We shall soon present to our readers another interesting paper from Dr. Gunning on his Splints for the Treatment of Fractures of the Lower Jaw, the electrotype plates of which are now being prepared.

